# Chiropractic conservatism and the ability to determine contra-indications, non-indications, and indications to chiropractic care: a cross-sectional survey of chiropractic students

**DOI:** 10.1186/s12998-018-0227-6

**Published:** 2019-02-19

**Authors:** Guillaume Goncalves, Marine Demortier, Charlotte Leboeuf-Yde, Niels Wedderkopp

**Affiliations:** 1 CIAMS, University of Paris-Sud, University of Paris-Saclay, F- 91405 Orsay Cedex, France; 2 CIAMS, University of Orléans, F-, 45067 Orléans, France; 3 Institut Franco Européen de Chiropraxie, 24 boulevard Paul Vaillant Couturier, F- 94200, Ivry sur Seine, France; 4 Institute for Regional Health Research, University of Southern Denmark, DK-5000 Odense, Denmark

**Keywords:** Étudiants en chiropraxie, Conservatisme, Subluxation, Contre-indication, Non-indication, Indication, Enquête, Chiropractic students, Conservatism, Subluxation, Contra-indication, Non-indication, Indication, Survey

## Abstract

**Background:**

While there is a broad spectrum of practice within chiropractic two sub-types can be identified, those who focus on musculoskeletal problems and those who treat also non-musculoskeletal problems. The latter group may adhere to the old conservative ‘subluxation’ model. The main goal of this study is to determine if chiropractic students with such conservative opinions are likely to have a different approach to determine *contra-indications*, *non-indications* and *indications* to chiropractic treatment versus those without such opinions.

**Method:**

An anonymous and voluntary survey on 3rd to 6th year French chiropractic students was conducted between November 2017 and January 2018. Level of chiropractic conservatism (10 items) and the ability to determine *contra-indications* (2 cases), *non-indications* (4 cases) and *indications* (3 cases) were evaluated through a questionnaire. Answers to these cases were dichotomized into ‘appropriate’ and ‘inappropriate’ answers, as defined by previous research teams and the present team. The level of conservatism was classified into four groups, ‘group 4’ corresponding to the highest score. Descriptive data are provided, and bi- and multivariate analyses were performed through logistic regression to test the associations between the level of conservatism and the ability to determine the suitability of chiropractic treatment.

**Results:**

In all, 359 of 536 (67%) students responded to the questionnaire. They generally recognized a number of *contra-indications* and *indications* to treatment. However, they found it more difficult to identify *non-indications*. The more conservative students were more likely to intend to treat their patients, even if this was irrelevant (*non-indications*). For example, those who were most conservative (group 4) were much more willing than those in group 1 to treat ‘chiropractically’ a 5-year-old child with no history of back pain or disease to prevent future back pain (OR = 14.7) and also to prevent non-musculoskeletal disease (OR = 22).

**Conclusion:**

It is concerning that students who adhere to the subluxation model are prepared to ‘operationalize’ their conservative opinions in their future scope of practice; apparently willing to treat asymptomatic people with chiropractic adjustments. The determinants of this phenomenon need to be understood.

**Electronic supplementary material:**

The online version of this article (10.1186/s12998-018-0227-6) contains supplementary material, which is available to authorized users.

## Abstract (fr)

**Introduction:**

S’il existe de nombreuses approches chiropratiques, deux types de chiropracteurs peuvent-être identifiés; ceux s’intéressant aux troubles musculo-squelettiques et ceux souhaitant prendre en charge aussi des troubles non musculo-squelettiques. Il est possible que ces derniers adhèrent au modèle conservateur de la *subluxation*. Le principal objectif de cette étude est de déterminer si les étudiants en chiropraxie ayant ce type d’opinions conservatrices ont une approche différente pour déterminer les *contre-indications*, *non-indications*, et *indications* au traitement chiropratique, comparés à ceux n’ayant pas ce type d’opinions.

**Méthode:**

Une enquête anonyme et volontaire sur les étudiants en chiropraxie de 3^ème^, 4^ème^, 5^ème^, et 6^ème^ années à l’Institut Franco Européen de Chiropraxie a été effectuée entre les mois de novembre 2017 et janvier 2018. Le niveau de conservatisme (10 items) et la capacité à déterminer les *contre-indications* (2 cas cliniques), les *non-indications* (4 cas cliniques) et les *indications* (3 cas cliniques) ont été évalués. Les réponses à ces cas cliniques ont été dichotomisées en réponses « appropriées » et « non appropriées », comme il avait été défini par les précédentes et l’actuelle équipe de recherche. Le niveau de conservatisme a été classé en quatre groupes, le score le plus élevé étant celui du groupe « 4 ». Les données descriptives ont été rapportées, des analyses bi- et multivariées ont été effectuées à travers des régressions logistiques. Le but étant d’évaluer s’il existe des associations entre le niveau de conservatisme et la capacité d’avoir une décision de prise en charge adaptée.

**Résultats:**

359 sur 536 étudiants (67%) ont répondu au questionnaire. Ils reconnaissent correctement quelques cas de *contre-indications* et d’*indications* au traitement chiropratique. Cependant, il leur est plus difficile de détecter les *non-indications*. Les plus conservateurs d’entre eux sont plus sujets à prendre en charge les patients, même si cela n’est pas nécessaire (*non-indications*). Par exemple, les plus conservateurs (groupe 4) sont plus enclins, comparés à ceux étant dans le groupe 1, à prendre en charge en chiropraxie un enfant de 5 ans n’ayant jamais eu de douleur ou maladies pour prévenir l’apparition de douleurs rachidiennes (OR = 14,7) et de maladies non musculo-squelettiques (OR = 22).

**Conclusion:**

Il est préoccupant de constater que les étudiants qui adhèrent au modèle de la *subluxation* soient prêts à intégrer ces opinions dans leurs futures prises en charge; souhaitant proposer des ajustements chiropratiques aux patients asymptomatiques. Les déterminants de ce phénomène se doivent d’être compris.

## Background

### Dual model in chiropractic: A scope of practice issue

In countries where chiropractic is regulated by law, it is generally accepted as a profession that deals deals with musculoskeletal conditions [1]. Therefore, chiropractors working in such jurisdictions, who also claim to prevent or treat non-musculoskeletal conditions, may break the law.

Chiropractors practice in several ways but one distinction relating to this issue is the separation between chiropractors who focus mainly on musculoskeletal disorders and those who state that they are unconcerned about patients’ presenting complaints, because they detect and remove ‘subluxations’ of the spine through chiropractic ‘adjustments’ [2]. These subluxations, it was claimed already a century ago, may have a detrimental effect on health [3] and their removal may, according to those beliefs, positively impact the prevention or recovery from many types of diseases, in addition to those of the musculoskeletal system [3]. Some chiropractors still adhere to this model [4, 5]. In this article, we shall call the first group of interest ‘musculoskeletal’ and the second group ‘conservative’. According to the Oxford dictionary, ‘conservatism’ is a commitment to traditional values and ideas with opposition to change or innovation [6]. This term does, therefore, in this article, not refer to a political conviction, it merely describes an approach to traditional chiropractic values. Although many ‘conservatives’ claim they are not treating illnesses directly, they will by definition be accepting patients who either wish to preserve good health or receive treatment for various non-musculoskeletal disorders. It is therefore fair to state that this group of chiropractors may deal with patients with a broader scope of conditions than the musculoskeletal group.

With regard to the subluxation (also called by a variety of other labels such as ‘fixation’) has not been shown to measurably exist and to our knowledge, there is no objective method to detect it before a spinal problem arises. In addition, according to a recent systematic critical review of the chiropractic literature which reviewed some research on the topic, there is no acceptable evidence supporting the concept that chiropractic adjustments can *prevent* the development of non-musculoskeletal disorders [7]. In fact, according to this systematic review, the only two articles of acceptable standard showed that this was not possible. Similarly, another review concluded that there is no evidence in favour of the successful *treatment* of non-musculoskeletal conditions using chiropractic methods [8]. The ‘conservatives’ are therefore pretending to treat something that is not easily captured and claiming to have an effect that has not been shown to occur.

This conservative approach was commonly accepted in the early years of chiropractic but it is not officially approved in modern chiropractic education standards. However, chiropractors have traditionally had a rather generous approach to whether the ‘conservatives’ should be allowed to practice in this way by accepting ‘fuzzy’ definitions and texts in order to accommodate both approaches. It is worth noting that the Standards for the Council on Chiropractic Education-International in 2010 [9], used a rather ‘generous’ definition of chiropractic, which reads: “The chiropractor, as a practitioner of the healing arts, […] must be well educated to diagnose, to care for the human body in health and disease and to consult with, or refer to, other health care providers when appropriate for best interest of the patient.”

However, in its latest revision, the World Federation of Chiropractic definition was used, which puts more emphasis on the musculoskeletal system by defining chiropractic as “a health profession concerned with the diagnosis, treatment and prevention of mechanical disorders of the musculoskeletal system, and the effects of these disorders on the function of the nervous system and general health” [10].

### Triage of patients: The understanding of contra-indications, non-indications and indications

For patients, it would likely matter if they consulted a musculoskeletal or a conservative practitioner, as these groups would manage their patients differently. Thus, we assume that these groups may not agree on *non-indications* for treatment. Examples of *non-indications* could be eczema, asthma, bedwetting, diabetes, ear infections and autism; conditions without an apparent biological rationale for chiropractic treatment but which normally would not likely worsen because of the chiropractic treatment. We postulate that chiropractors who are convinced that the subluxation model is correct are likely to assume that chiropractic treatment is inherently valuable and are therefore willing to accept patients with a multitude of disorders, on the understanding that they are entitled to do this because they are treating only the spine. The musculoskeletal practitioner, on the other hand, is less likely to accept patients with non-musculoskeletal diseases. Therefore, most types of non-musculoskeletal disorders can be classified as *non-indications* for chiropractic prevention or treatment by musculoskeletal practitioners. Consequently, the list of *indications* is likely to differ for these two types of chiropractors.

However, because of their training in differential diagnosis it is our opinion that both groups of chiropractors are likely to identify correctly *contra-indications* to treatment. *Contra-indications* can be defined as conditions that could worsen with spinal adjustments (such as severe osteoporosis or an aortic aneurysm).

### Prevention aspects of the dichotomous chiropractic approach

Primary prevention is defined as prevention of a condition before it has occurred [11]. According to a recent systematic review of the literature, chiropractors are generally interested in providing primary prevention to their patients, both in relation to non-musculoskeletal and musculoskeletal disorders. An example is advising their patients to have a healthy lifestyle [12]. This model of care is, in our opinion, both reasonable and logical. However, there is no evidence that chiropractic adjustments per se can prevent non-musculoskeletal conditions, as in primary prevention, and there is no evidence that they can prevent future diseases [7]. Therefore, to offer chiropractic treatment/adjustments to primarily prevent either musculoskeletal or non-musculoskeletal problems, idealistic as it may be, is based only on aspiration and personal opinion.

Secondary prevention is defined as early treatment of disease so as to prevent its continuation, and tertiary prevention is described as treatment of the chronically ill, to maintain their status at a reasonable level or to prevent further deterioration [11]. Both secondary and tertiary prevention of back pain should be relevant to the chiropractic profession, as musculoskeletal problems often are episodic or chronic [13]. Chiropractors have long believed this and have attempted to improve the quality of life for patients with recurring back problems, by means of so-called ‘maintenance care’. The percentage of chiropractors using this approach has been shown to vary greatly, such as between 2 and 95% of Swedish chiropractors’ patients belonging to this category [14] and between 0 and 100% of Danish chiropractors’ patients [15]. But a closer look at how it is used reveals that there is reasonable consensus among chiropractors that its indications are i) a certain number of previous episodes of low back pain (LBP) ii) in patients who respond well to chiropractic treatment [15]. Not only does maintenance care in a recurring musculoskeletal disorder seem logical, but it has also been shown in a large randomized controlled multicentre clinical trial, using the above inclusion criteria that this type of patients, had a considerably better outcome if they received maintenance care than those who received care only when they felt they needed it [16]. Thus, this type of treatment approach, so far, seems to have the best documented effect in chiropractic practice as compared to the usual treatment.

In other words, primary prevention of both musculoskeletal and non-musculoskeletal conditions through chiropractic adjustments could be considered *non-indications*, whereas maintenance care in patients with a history of episodic low back pain and good outcomes with chiropractic treatment would be an *indication*. On the other hand, maintenance care should not be offered to all patients who happen to consult a chiropractor as there is no obvious rationale for such an approach and no evidence for a general effect.

### Chiropractic students and their ability to recognize contra-indications, non-indications and indications to treatment

A recent study of chiropractic students in Australia [17] revealed that they generally found it more challenging to detect *non-indications* than *contra-indicated* and *indicated cases*. Interestingly, studies have shown that also present-day chiropractic students may cling to the subluxation model and that this can also occur in institutions that do not adhere to that type of approach. Thus, approximately half of the students in this Australian study (from Murdoch University and Macquarie University) erroneously thought that chiropractic spinal adjustments can help the immune system or improve the health of infants. Further, approximately three quarters of students were of the opinion that chiropractic spinal adjustments can prevent degeneration of the spine and also help the body to function at 100% of its capacity [18].

The question arises, do chiropractic students with such attitudes have a different approach to *contra-indications*, *non-indications* and *indications* to chiropractic treatment versus those who do not have this strong confidence in the power of the chiropractic adjustment? To answer this question and obtain more information on this topic, a survey was carried out on chiropractic students in years 3 to 6 at the *Institut Franco Européen de Chiropraxie* (at its two campuses in Toulouse and Paris, France)*.* This is a European Council on Chiropractic Education-accredited undergraduate institution with a musculoskeletal approach, as regulated by the French Government [19], existing in a country where chiropractic has been legally recognized since 2002 [20].

The main goal of this cross-sectional survey on French chiropractic students was to investigate if students’ attitudes and opinions on various chiropractic concepts and their psychological profile could help explain their future clinical approach. The present report deals with chiropractic students’ ability to relate logically to the concept of triage and the potential influence that various degrees of chiropractic conservatism in relation to the subluxation model can have on this ability.

#### Our research questions were

1. What is the ability of chiropractic students to determine *contra-indications*, *non-indications*, and *indications* to chiropractic care in relation to

- primary prevention?
- initial course of treatment?
- long term strategies?

2- Do these triage abilities differ with academic year of study?
3- Is there a link between students’ attitudes to the ‘subluxation model’ and their ability to determine *contra-indications*, *non-indications* and *indications* to treatment?

## Methods

### Ethics

The study was approved by the Ethics Committee of the University of Paris-Saclay (File no: 2017/11).

### Settings, study participants and data collection

This anonymous and voluntary survey was conducted on chiropractic students in the 3rd to 6th years of study at the *Institut Franco-European de Chiropraxie* at its two sites in Toulouse and Paris, in France. Information was sent to all students by e-mail, and oral information was provided in class before handing out the questionnaires that were completed during a normal lecture and given back independently of the researchers. The time needed to fill out the questionnaire was approximately 45 min. The first sessions took place in November/December 2017 after an invitation by e-mail. Two additional sessions were organized in January 2018 for those who were absent at the first session. These students were invited by e-mail to participate in this extra session, having been identified as previously absent through the roll call. Their responses were also anonymous.

### The survey instrument

The survey instrument consisted of material for two separate studies. In the present report two questionnaires were included on treatment strategies, with some questions on ‘subluxation’ (*n* = 4), chiropractic ‘adjustments’ (*n* = 6), and primary prevention for a 5 yr. old child (*n* = 2). The second study will be reported elsewhere.

#### Questionnaires on treatment strategies

##### Low back pain questionnaire

We used a questionnaire consisting of nine clinical cases on low-back pain with a number of possible answers [14]. It had been previously validated in an interview study [21], which showed that participants had understood the questionnaire and that their responses were similar to those in a previous survey. The questionnaire has thereafter been used in France [22], which largely confirmed the previous profiles and on a student population in Australia [23]. In the previous study the questionnaire (originally in English) had been translated into French and back translated into English, [22] and we used that version.

##### Neck pain questionnaire

We also included also a questionnaire consisting of five clinical cases on neck pain, used in a study on French chiropractors [24], in which answers were found to be essentially coherent and logical. This survey was previously used in a chiropractic student population in Australia [23], with logical answers.

##### Separate clinical cases created for this survey

Two clinical cases on primary prevention of a 5-year-old child were created by the present research team directly in French. These two cases are available in Additional file 1.

#### Questionnaire on conservatism

##### Separate items taken from other studies

Seven additional items came from different studies. One item related to the concept of the ‘subluxation’ [25], the others on chiropractic ‘adjustments’ [23, 25]. These items were translated into French (back translation English/French – French/English) by two bilingual individuals unfamiliar with the questionnaire and without any communication between the two translators.

##### Separate items created for this survey

Three items on ‘subluxation’ were created by the present research team directly in French.

#### Additional collected data not included in the present report

In addition, but not dealt with in the current report, there were two brief psychological questionnaires and some items relating to self-confidence, the future use of prescriptive chiropractic techniques, and knowledge, attitudes and opinions of/about Functional Neurology, a specific chiropractic treatment system [26]. An additional psychological questionnaire was included in the survey but because of a clerical error, some of the text went missing and was therefore not incorporated in any of the studies.

#### Pilot study

A pilot study was conducted of the entire survey with at least one of the authors present to be able to discuss problems and comments on the questionnaire. Participants were eight former students, who had passed their final exams but still attended the clinic. This resulted in a few minor language changes to facilitate the comprehension of the whole survey.

### Variables of interest and their rationale

From this survey, some variables were selected in addition to site, year of study, grade, and sex of respondent.

- Independent (predictor) variable: ten items were used to evaluate the level of chiropractic conservatism of the students in relation to the subluxation model, making it possible to score in total between 0 and 10. These items dealt with the opinions of chiropractic students about chiropractic ‘adjustments’ (*n* = 6) and their beliefs in the ‘subluxation’ (*n* = 4) (Additional file 1).
- Nine dependent variables were selected in relation to acceptance of treatment, four from the low back pain questionnaire, three from the neck questionnaires and two from additional independent questions. There were two *contra-indicated* cases, four *non-indicated* cases and three *indicated* cases. The ‘appropriate’ vs. ‘inappropriate’ answers proposed in the previous study on this topic for the seven low back and neck questions were used [23]. Concerning the two additional questions, the members of the present research team decided which treatment choices were ‘appropriate’ or not. The questions are presented in Additional file 1.

The rationale for the ‘appropriate’ answers to clinical cases are presented in Additional file 2, and the description of the conservatism items is given in Additional file 3.

### Data management and analysis

Data were entered in EPIDATA 3 twice by the first two authors; first with one reading from the pre-coded questionnaires and the other entering the information, to thereafter check the entered data by switching roles. All analysis were done in STATA 15.

#### Transformation of data

The ten items relating to attitudes to the subluxation model, all with the five answer possibilities, were dichotomized into ‘appropriate’ answers (0 point) and ‘inappropriate’ answers (1 point) (Additional file 1).

A conservatism score was created by adding up the ‘inappropriate’ answers, placing them in four groups: group 1 (scores 0–2); group 2 (scores 3–5); group 3 (scores 6,7) and group 4 (scores 8–10) based on the distribution of data and common sense. As very few students scored ‘0’, we considered that it would be possible to accept one or two ‘subluxation’ statements without being a hard-core conservative, for which reason we also included also the scores of 1 and 2 in the lowest group.

The questions on the various types of indications for or against treatment had five to seven answering possibilities. Also, these were dichotomized into ‘appropriate’ and ‘inappropriate’ answers.

All these transformations are shown in Additional file 1.

#### Bi- and multivariate analysis

The associations between the independent variable (level of conservatism) and dependent variables (*contra-indications*, *non-indications* and *indications*) were tested for statistical significance using logistic regression, reported as odds ratios (OR) with their 95% confidence intervals (CI), after which the analyses were repeated, controlled for site, sex, and year of study. When CIs did not overlap, differences between groups were considered statistically significant. Results have been presented as exact estimates in the tables and summarized in the text, for ease of understanding.

## Results

### Descriptive information

In all, 359 of 536 students (67%) returned the questionnaire, of which 241 (67%) were females; 160/199 students (80%) in Toulouse and 199/337 (59%) in Paris. The distribution of responders and non-responders in relation to site (Toulouse or Paris), sex, and year of study (3rd to 6th) is shown in Table 1. The descriptive variables (site, sex, and year of study) and predictor variable (conservatism score) are presented in Table 2. Also included in this table are the responses relating to this score that have been grouped into the four overall categories with ‘group 4’ indicating the most conservative approach.

**Table 1:** Response rates for participation in a survey of 359 French chiropractic students

Year of Program	Location	Males	Females	% of respondents by year
		(% of responders by sex)		
6th year	Toulouse	8 (61%)	27 (77%)	73
	Paris	17 (55%)	34 (65%)	59
5th year	Toulouse	13 (81%)	23 (88%)	86
	Paris	11 (33%)	25 (44%)	40
4th year	Toulouse	15 (79%)	37 (92%)	90
	Paris	15 (45%)	32 (58%)	53
3rd year	Toulouse	10 (62%)	27 (79%)	74
	Paris	29 (80%)	36 (90%)	85

**Table 2:** Descriptive table of independent/predictor variables in a survey of 359 French chiropractic students

Variables	*N* (%)
Descriptive variables	
Site	
- Toulouse	160 (45)
- Paris	199 (55)
Sex	
- Males	118 (33)
- Females	241 (67)
Year of study	
- 6th year	86 (24)
- 5th year	72 (20)
- 4th year	99 (28)
- 3rd year	102 (28)
Predictor variable: conservatism score	
Score	
0	3 (1)
1	7 (2)
2	5 (1)
3	16 (4)
4	25 (7)
5	29 (8)
6	42 (12)
7	76 (21)
8	81 (23)
9	63 (18)
10	7 (2)
Non response to all of the items	5 (1)
Group 1 (scores 0–2)	15 (4)
Group 2 (scores 3–5)	70 (20)
Group 3 (scores 6, 7)	118 (33)
Group 4 (scores 8–10)	151 (42)
Non response to all of the items	5 (1)

### Ability to determine contra-indications

**Fig. 1:**
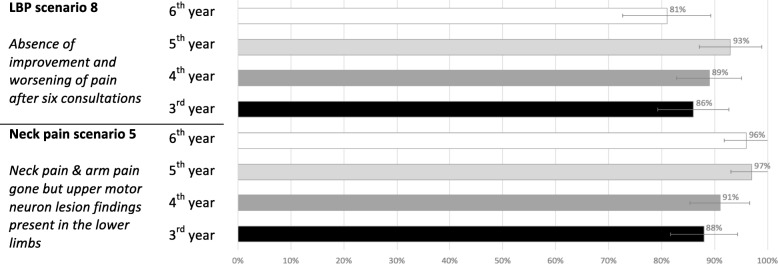
Proportion of chiropractic students able to select contra-indications for chiropractic treatment

The ability to detect correctly the two *contra-indications* is shown in Fig. 1, separately for each year. The vast majority of the students (between 81 and 97%) could detect the two cases of contra-indications; one describing a patient who had motor neuron lesion findings in the lower limbs and the other a case whose LBP worsened after six consultations.

### Ability to determine non-indications

**Fig. 2:**
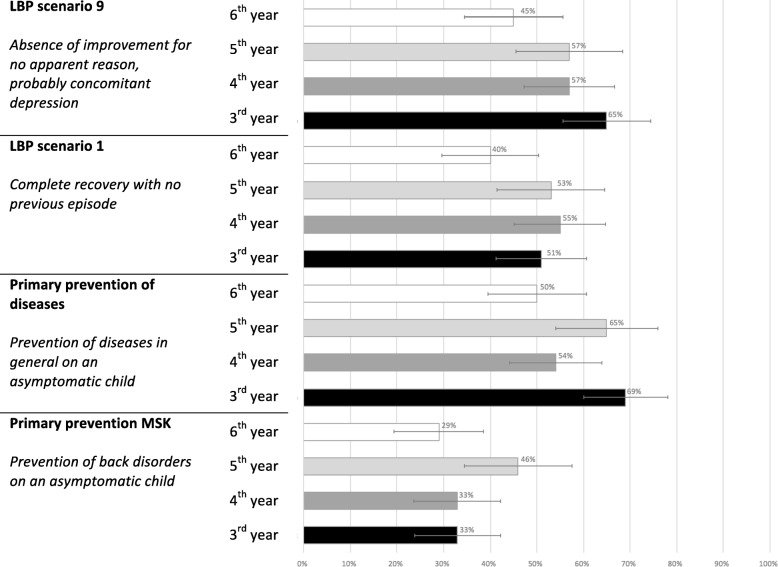
Proportion of chiropractic students able to select non-indications for chiropractic treatment

As can be seen in Fig. 2, the results for the *non-indications* were considerably lower than for the *contra-indications.* In general, *non-indications* to treatment were recognised by approximately only half of the responders considering (i) absence of improvement in a probably depressed patient (between 45 and 65%), (ii) complete recovery in a person with no previous episodes (between 40 and 55%), and (iii) prevention of future diseases in general on an asymptomatic child (between 50 and 69%). However, the lowest estimates of acceptable answers (between 29 and 46%) were found for the case of the prevention of spinal pain in an asymptomatic child.

### Ability to determine indications

**Fig. 3:**
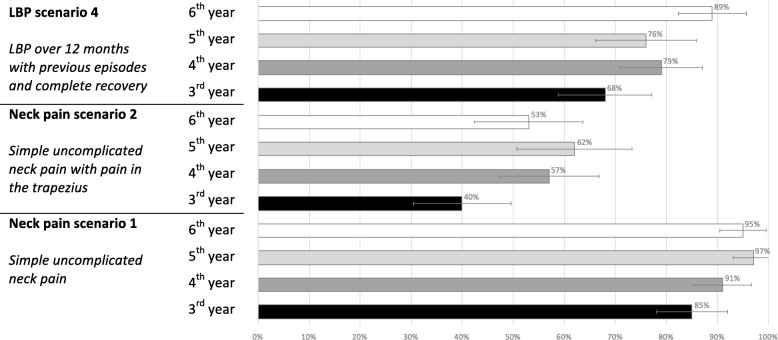
Proportion of chiropractic students able to select indications for chiropractic treatment

The ability to identify correctly *indications* to treatment by year of study is shown in Fig. 3. For the low back pain scenario, the vast majority of students, regardless of year of study, considered it an indication for treatment (between 68 and 89%). The same was noted for a simple case of neck pain (between 85 and 97%). However, when the neck pain in the previous case was complicated by pain in the Trapezius muscle, there were significantly fewer acceptable replies (between 40 and 62%).

### Links between students’ attitudes to the ‘subluxation model’ and their ability to determine contra-indications, non-indications and indications

The non-adjusted and adjusted analyses for the influence of various degrees of conservatism on clinical decisions are shown in Tables 3 and 4. There were no obvious associations between the degree of conservatism and the ability to identify *contra-indications*. The odds ratios were close to 1, and the confidence intervals included 1, thus statistically insignificant.

**Table 3:** Non-adjusted associations between level of conservatism and clinical inability to determine contra-indications, non-indications and indications

Conservatism groups	Contra-indications		Non-indications				Indications		
	Neck pain scenario 5 OR [95% CI]	LBP scenario 8 OR [95% CI]	Primary prevention of back disorders OR [95% CI]	Primary prevention diseases in general OR [95% CI]	LBP scenario 1 OR [95% CI]	LBP scenario 9 OR [95% CI]	Neck pain scenario 1 OR [95% CI]	Neck pain scenario 2 OR [95% CI]	LBP scenario 4 OR [95% CI]
1 (index)	1	1	1	1	1	1	1	1	1
2	1.7 [0.2–14.6]	0.5 [0.1–2.9]	3.4 [0.9–13.0]	3.4 [0.4–28.4]	1.7 [0.5–6.0]	0.7 [0.2–2.1]	1.0 [0.2–5.0]	0.5 [0.1–1.5]	0.4 [0.1–1.1]
3	1.0 [0.1–8.3]	1.0 [0.2–5.0]	8 [2.1–30.1]	7.9 [1.0–62.1]	2.5 [0.7–8.3]	0.6 [0.2–1.8]	0,5 [0.1–2.5]	0.7 [0.2–2.1]	0.1 [0.0–0.4]
4	1.3 [0.2–10.8]	1.2 [0.2–5.5]	13.8 [3.7–51.7]	20.4 [2.6–158.8]	4.3 [1.3–14.1]	0.7 [0.3–2.2]	0,5 [0.1–2.6]	0.3 [0.1–0.9]	0.1 [0.0–0.4]

*CI*=Confidence interval

**Table 4:** Adjusted associations between level of conservatism and clinical inability to determine contra-indications, non-indications and indications

Conservatism groups	Contra-indications		Non-indications				Indications		
	Neck pain scenario 5 OR* [95% CI]	LBP scenario 8 OR* [95% CI]	Primary prevention of back disorders OR* [95% CI]	Primary prevention of diseases in general OR* [95% CI]	LBP scenario 1 OR* [95% CI]	LBP scenario 9 OR* [95% CI]	Neck pain scenario 1 OR* [95% CI]	Neck pain scenario 2 OR* [95% CI]	LBP scenario 4 OR* [95% CI]
1 (index)	1	1	1	1	1	1	1	1	1
2	1.2 [0.4–3.2]	0.6 [0.1–3.7]	3.5 [0.9–13.5]	3.9 [0.5–33.0]	1.7 [0.5–6.4]	0.8 [0.3–2.2]	0.8 [0.2–4.3]	0.5 [0.1–1.6]	0.4 [1.1–1.3]
3	0.7 [0.3–1.7]	1.2 [0.2–6.6]	8.3 [2.2–31.5]	9.0 [1.1–72.2]	2.5 [0.7–9.0]	0.7 [0.2–1.8]	0.4 [0.1–2.1]	0.7 [0.2–2.2]	0.2 [0.1–0.5]
4	1 (omitted)	1.4 [0.3–7.1]	14.7 [3.8–56.6]	22.0 [2.7–175.6]	4.6 [1.3–16.2]	0.7 [0.3–2.0]	0.5 [0.1–2.5]	0.3 [0.1–1.0]	0.2 [0.1–0.6]

*CI*=Confidence interval *adjusted for site, sex, and year of study

For the *non-indications*, however, the odds ratios increased in a dose-response fashion to reach 13.8 [95% CI: 3.7–51.7] (primary prevention of back disorders in a child); 20.4 [2.6–158.8] (primary prevention of disease in general in a child), and 4.3 [1.3–14.1] (maintenance care in a patient who recovered completely from simple LBP with no previous episode). These results are shown in Table 3. Because of the relatively small study sample, the confidence intervals were sometimes large. In a case of LBP with no improvement for no apparent reason, probably with concomitant depression, there was no such association with the degree of conservatism. When controlling for site, sex, and study year (Table 4), no obvious changes occurred in these estimates.

For the *indications* of treatment, the estimated odds ratios were lower than 1 (i.e. ‘protective’), significantly so in three cases (Table 3 for the non-adjusted values). After multivariate analysis, two of these estimates remained statistically significant, indicating that the two groups that held the strongest conservative views were 80% less likely to *not* identify the need for maintenance care in the case of a person who had experienced LBP for 12 months with previous episodes and complete recovery after treatment (Table 4). In other words, the OR = 0.2 [0.1–0.6] indicates that the highest conservative score (group 4) had a ‘protective’ effect against giving the ‘wrong’ answer to this question, i.e. good at recognising the indicated case.

### Comparison between years of study

Most estimates increased with year of study for the *contra-indicated* and *indicated* cases, but these differences were not significant. On the other hand, for the *non-indicated* cases, these estimates are reversed and the proportion of 6th year students with correct answers was always less than in the lower years. Again, these differences were not significant.

### Summary in relation to asymptomatic patients

In sum, four of the nine clinical cases related to asymptomatic patients. In all these, the more conservative students indicated that they would be prepared to treat, regardless of the motive of consultation (musculoskeletal or non-musculoskeletal) and history of the complaint (first episode or recurrent LBP).

## Discussion

### Abilities of chiropractic students to select suitable patients

According to this survey, French chiropractic students in their 3rd to 6th years of study can recognize a number of *contra-indications* and *indications* to treatment. However, they found it more difficult to identify *non-indications*, as only half of them got the answers correct on three of these items. Moreover, even fewer students (generally less than 40%) considered primary prevention for future back pain problems to be an unsuitable indication for treatment. Interestingly, the lowest estimates were always found in the 6th year of study.

This pattern was very similar to a recent study of Australian chiropractic students [17], but French chiropractic students were better at identifying a *contra-indication* in the case of worsening LBP after six visits. On the other hand, the Australian students were better at identifying a case of neck pain radiating to the trapezius muscle, as an *indication* for treatment.

### Conservatism and the ability to perform triage of patients

To our knowledge, this is the first study to compare chiropractic students’ ability to determine when various presented cases would be *contra-indicated* to treatment and their ability to distinguish between *non-indications* and *indications* to treatment, in relation to their tendencies toward conservatism. There was an association between conservatism and the inability to detect *non-indications* and this association increased with the level of conservatism, as measured by our score based on ten items relating to the subluxation model. Interestingly, the results did not improve closer to graduation.

The inability of the conservative students to detect *non-indications* is logical, as the subluxation model implies that patients can be treated, more or less, regardless of symptoms or the reason for the consultation. As the consequence of the subluxation model is an almost unlimited scope of practice, the findings also indicate that, in some instances, students are potentially going to practice outside the legal boundaries of French law relating to the chiropractic profession [27].

### Chiropractic conservative students intend to treat asymptomatic patients

Modern concepts in back pain were discussed recently by a multi-professional group of experts, including chiropractors, in a series of articles in the Lancet, in which emphasis was put on the necessity to stop useless treatments for back pain [28, 29]. From this perspective, it is inappropriate to treat asymptomatic people in order to seek to prevent, for example, non-musculoskeletal diseases. Further, it is not in accordance with the main motive for consultation in chiropractic practice (which is musculoskeletal conditions) [30]. Also, although the primary prevention of musculoskeletal disorders through chiropractic treatment may feel intuitively correct for many chiropractors, at this time there is no evidence that this is possible.

## Methodological considerations

The questionnaire was anonymous and voluntary. The response rate of this study was 67%, which we consider a relatively acceptable result, but since the survey was carried out anonymously we could not confirm generalisability to the entire student population of IFEC by comparing responders to non-responders. For this reason, we do not know if the presence of the non-responders could have improved or worsened the results. Since our results were similar to those of the previous Australian studies [17, 18], we assume that our results are probably valid.

The outcome variables were selected from two previously used and, in one case, validated questionnaire. Our items relating to the subluxation model were mainly selected from previous studies but some were designed by the authors. We believe that these questions cover fairly well the concepts frequently held by this group of chiropractors. Further, the user-friendliness of the questionnaire was tested in a pilot study with only a few modifications needed. The time required to fill out the entire questionnaire was approximately 45 min, which would be sufficient for a group of students who are used to reading and participating in intellectual activities over prolonged periods. This was confirmed by the low number of missing data.

### Educational perspectives

Our results were similar to those of a recent survey from Australia, which showed that a large proportion also of their chiropractic students adhere to similar concepts and have problems selecting the correct type of patient for treatment [17, 18]. What our study added was a confirmation of these findings and the knowledge that the sub-group of students with a conservative approach to chiropractic adhere to the concept of a broader scope of practice. This is concerning and the realisation that these two student populations (in Australia and in France) were so similar evokes the suspicion that this may be a more widespread phenomenon possibly to be found in other institutions. Interestingly, the attitudes to non-indications did not improve with ‘year of study’. Therefore, the educational approach in relation to both the history of chiropractic and clinical topics need to be revisited.

Also, our results were not dependant on site or year of study. It is therefore possible that external practitioner influences were at play, or some of the lecturers influenced the students in this direction, contrary to the philosophy and policy of the institution. However, the authors are well acquainted with the attitudes of most staff on this issue and doubt that this is the case. Also, there are similarities with two completely different study programs taking place in Australia. All these three education programs have a musculoskeletal approach. Therefore, the causes should perhaps be sought from within the student group, as this type of conservatism is not encouraged within these institutions. Explanations could perhaps be that young people with certain personality traits are attracted to the chiropractic program and that such students are fascinated by more ideological movements. An idealistic approach to a broad scope of practice is probably also more common in young people without any real clinical experience. The influence of guest lecturers and ‘fringe’ chiropractors circulating the schools with a hidden curriculum could perhaps also explain some of this finding. Thus, it is possible that various subcultures may develop among students unbeknownst to the schools. This may counteract the significant efforts to provide the students with a modern view of chiropractic within the legal boundaries of the profession, as it is probably defined in many countries.

### Research perspective

Given the demands put upon modern chiropractic in those countries where this profession enjoys legal status, it would be relevant to identify the causes of this strong conservative movement among students. Remedial activities could be undertaken, including different pedagogical approaches based on such information, with a need to be monitored, and a long-term strategy put in place to come to terms with this unfortunate finding.

## Conclusion

Chiropractic students are able to recognize *contra-indications* and *indications* but find it more challenging to identify *non-indications* in chiropractic clinical cases. Moreover, students who adhere to a conservative chiropractic approach systematically wish to treat patients, regardless of the symptoms, and even if they present with *non-indications*. The apparent presence of the conservative approach is of concern because it may predict a proportion of our future chiropractors scope of practice. Therefore, the determinants of this phenomenon need to be explored and understood.

## Additional files

Additional file 1: Questionnaire used in a survey on French chiropractic students. (DOCX 731 kb)

Additional file 2: Rationale for ‘appropriate answer’ to clinical cases given to French chiropractic students. (DOCX 23 kb)

Additional file 3: Rationale for choice of items relating to conservative approaches included in a survey of 359 French chiropractic students. (DOCX 32 kb)

## Abbreviations

CI: Confidence Interval
LBP: Low Back Pain
OR: Odd Ratio
