# Supplementary material for: Chiropractic conservatism and the ability to determine contra-indications, non-indications, and indications to chiropractic care: a cross-sectional survey of chiropractic students
Source: Chiropr Man Therap. 2019 Feb 19;27:3. doi: 10.1186/s12998-018-0227-6 (PMC6379950; doi:10.1186/s12998-018-0227-6)
Supplement: Supplementary file 1 — Questionnaire used in a survey on French chiropractic students. (DOCX 731 kb) [file 12998_2018_227_MOESM1_ESM.docx]

**Additional File 1. Questionnaire used in a survey on French chiropractic students**

|  | Definitely not | Probably not | Don’t know | Yes, probably | Yes, definitely |
| --- | --- | --- | --- | --- | --- |
| **In your opinion, can chiropractic spinal adjustments** |  |  |  |  |  |
| prevent disease in general? [1] | Appropriate  answer | | | Inappropriate answer | |
| help the immune system? [1] |  |  |  |  |  |
| improve the health of infants? [1] |  |  |  |  |  |
| help the body function at 100% of its capacity? [1] |  |  |  |  |  |
| prevent degeneration of the spine? [1] |  |  |  |  |  |
| **Three clinical cases** | | | | | |
| Primary prevention of back disorders  A mother wants to bring her 5-yr. old child for regular chiropractic consultations to prevent the onset of spinal disorders in the future. The child has never had back pain before. Are you willing to regularly adjust this child to avoid the onset of back disorders in the future? [Created by the authors in French] | Appropriate  answer | | | Inappropriate answer | |
| Primary prevention of diseases  A mother wants to bring her 5-yr. old child for regular chiropractic consultations to prevent the onset of disease in the future. The case history reveals many diseases in the family (breast cancer, diabetes, lipidaemia, etc.). Are you willing to regularly adjust this child to avoid the onset of disease in the future? [Created by the authors in French] |  |  |  |  |  |

| For each statement, choose the box that best corresponds to your opinions | Strongly disagree | Somewhat disagree | I don’t know | Somewhat agree | Strongly agree |
| --- | --- | --- | --- | --- | --- |
| Subluxations are the cause of all disease [2] | Appropriate  answer | | | Inappropriate answer | |
| Subluxations cause short-circuits of the nervous system [Created by the authors in French] |  |  |  |  |  |
| Subluxations can have a negative effect on the capacity of the nervous system to provide energy to tissues and organs [Created by the authors in French] |  |  |  |  |  |
| It is possible to detect subluxations before symptoms appear [Created by the authors in French] |  |  |  |  |  |
| It is appropriate for every person to receive chiropractic adjustments for their entire life [2] |  |  |  |  |  |

**Cervical clinical cases**

A 28-year old man, tennis player by profession, consults you for a right-sided intense neck pain without any radiating pain. You note an antalgic position of the head, no other musculoskeletal signs (no torticollis), no other health problems in particular, normal x-rays for his age, and there are no red flags.

In each of the cases described below, what would you do?

|  | | | I would treat the patient on my own | I would treat the patient with the assistance of another paramedic ^(1)^ | I would treat the patient with the assistance of a general practitioner | I would treat the patient whilst asking the opinion of a specialist ^(2)^ | I would not treat the patient but refer him out | | Other, please explain page 9 ^(3)^ |
| --- | --- | --- | --- | --- | --- | --- | --- | --- | --- |
|  | 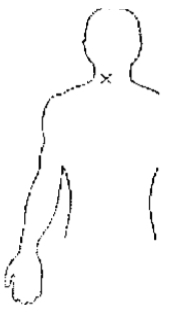 | Neck pain scenario 1  Physical examination: very tense cervical musculature, no neuro-vascular problems, right C5-6 painful on palpation, pain 7/10 on a visual analogue scale | Appropriate  answer | Inappropriate answer | | | | | |
|  | 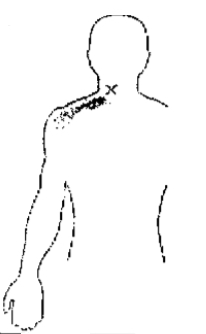 | Neck pain scenario 2  Five days later the patient comes back to you: same clinical signs but the pain now radiates into the right shoulder | Appropriate  answer | Inappropriate answer | | | | | |
|  | 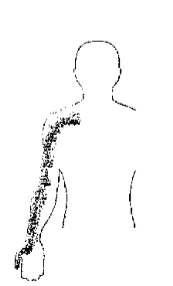 | Neck pain scenario 5  Ten days later: The symptoms and signs are the same as last time but in addition the following is noted: the right leg shows hyperreflexia (graded as 3+), a positive  sign of Babinski on the right and slight hypoaesthesia of the right leg | Inappropriate answer | | | | Appropriate  answer | Inappropriate answer | |

1. In this study, these professions are considered to be paramedics: physiotherapist and occupational therapist only.
2. In this study, these professions are considered to be specialists: neurologist, rheumatologist and surgeons only.
3. If you decide to refer to another health care professional, please note this in the box “other”, and explain it on page 9.

**Low back pain (LBP) clinical cases**

A 40-year-old man who consults with you for low back pain with no additional spinal or musculoskeletal problems, and with no other health problem.

His x-rays are normal for his age. There are no red flags.

The case above could proceed in the **following 9 ways** described on the next page.

For each of these nine possibilities, you will find six different strategies (A – F) and one possibility to define your own strategy (G). In this case, you will be able to detail it on page 12.

| A | **Second opinion** | I would refer the patient to another health care practitioner for a second opinion. |
| --- | --- | --- |
| B | **External help, keep in touch** | I would advise the patient to seek additional treatment whilst following the patient. |
| C | **Quick-fix** | I would tell the patient that the treatment is completed but that he is welcome to make a new appointment if the problem returns. |
| D | **Try again** | I would not consider the treatment to be fully completed and would try a few more treatments and perhaps change my treatment strategy, until I am sure that I cannot do any more. |
| E | **Symptom guided maintenance care** | I would follow this patient for a while, attempting to prolong the time period between visits until either the patient is asymptomatic or until we have found a suitable time lapse between check-ups to keep the patient symptom-free. |
| F | **Clinical findings-guided maintenance care** | I would recommend that the patient continues with regular visits, as long as clinical findings indicate treatment (e.g. spinal dysfunction/subluxation), even if the patient is symptom-free. |
| G | Other | None of the above (Please explain on page 12 in legible handwriting). |

**Scenarios included in this report:**

LBP scenario 1

An acute attack of LBP of 2 days’ duration and no previous history of LBP. The pain is completely gone after 2 visits. The patient seems to be an uncomplicated person and capable to look after himself and his back.

**C = appropriate answer**

LBP scenario 4

An acute attack of LBP of 1 week’s duration. The patient has had several similar attacks over the past 12 months. The pain is completely gone after 2 weeks of treatment.

**E and F = appropriate answers**

LBP scenario 8

The patient has had LBP intermittently over the past year. After the 2^nd^ visit the pain was 20% better, but today, after 6 visits, the patient has got gradually worse.

**A = appropriate answer**

LBP scenario 9

The patient has had LBP intermittently over the past year. After 6 visits the pain is 20% better. The symptoms come and go for no apparent reason. The patient appears tired and moody.

**A = appropriate answer**

**REFERENCES**

1. Innes SI, Leboeuf-Yde C, Walker BF: **The relationship between intolerance of uncertainty in chiropractic students and their treatment intervention choices**. *Chiropr Man Therap* 2017, **25**:20.

2. Gliedt JA, Briggs S, Williams JS, Smith DP, Blampied J: **Background, expectations and beliefs of a chiropractic student population: a cross-sectional survey**. *J Chiropr Educ* 2012, **26**(2):146-160.
