# Supplementary material for: Chiropractic conservatism and the ability to determine contra-indications, non-indications, and indications to chiropractic care: a cross-sectional survey of chiropractic students
Source: Chiropr Man Therap. 2019 Feb 19;27:3. doi: 10.1186/s12998-018-0227-6 (PMC6379950; doi:10.1186/s12998-018-0227-6)
Supplement: Supplementary file 3 — Rationale for choice of items relating to conservative approaches included in a survey of 359 French chiropractic students. (DOCX 32 kb) [file 12998_2018_227_MOESM3_ESM.docx]

**Additional file 3. Rationale for choice of items relating to conservative approaches included in a survey of 359 French chiropractic students**

The items included in this survey were selected from two angles; to understand students opinions on the indications for use of chiropractic spinal adjustments and their mechanism of action, although to some degree these dimensions overlap. Included is also a question on the concept that chiropractors can always detect a ‘subluxation’ also in the absence of disease and symptoms. Here we explain why we think that these items relate to a scientifically outmoded approach to chiropractic practice.

The answering possibilities are available in Additional file 1.

1. Questions about the indications for use of chiropractic spinal adjustments

**In your opinion, can chiropractic spinal adjustments …**

- **prevent disease in general?**
- **help the immune system?**
- **improve the health of infants?**
- **help the body function at 100% of its capacity?**

These four statements lack a scientific rationale and are not supported by the scientific literature. For example, in a review by Bronfort et al [1], 12 non musculoskeletal conditions were scrutinized for effect of treatment but there was no ‘high’ level of evidence for any of these. In relation to the ‘moderate’ evidence found, four disorders (i.e. asthma, infant colic, dysmenorrhoea, and stage 1 hypertension) were all negative. For the other eight the evidence was always inconclusive.

An updated review of the previous one [2] reported no additional ‘high’ level evidence, and there was no new ‘moderate’ evidence in a positive direction. In fact, the previous evidence was reduced in number because of additional studies (from four to two of ‘moderate’ but negative evidence). All other results were grouped under ‘inconclusive’. In sum, there was no evidence in favour of the treatment of non-musculoskeletal conditions.

In relation to primary prevention, a recent systematic review [3] shows that there are no studies of robust design to support a positive effect of primary prevention and early secondary prevention of diseases through chiropractic treatment. Only two clinical studies with a control group were found and these showed there to be no effect.

- **prevent degeneration of the spine?**

If chiropractic treatment can improve the mechanisms of the spinal structures it would seem logical that this could lessen the wear and tear. However, it has never been shown that spinal adjustment has such a beneficial effect, only that it often seems to lessen pain and discomfort.

Further, degeneration of spinal tissues is a process predetermined by nature (genetic) [4] and also influenced by environmental factors. Factors such as hard work and anatomical features are more dominant in life than chiropractic adjustments, hence it is unlikely that the chiropractic treatment would have a stronger effect than Mother Nature.

**It is appropriate for every person to receive chiropractic adjustments for their entire life**

Again, there is no rationale for such claim. Pretending this would mean that there is no *non-indication* to chiropractic treatment and that the chiropractic adjustment in some mysterious way is inherently beneficial for all people at all times.

1. Questions about subluxations

**Subluxations …**

- **are the cause of all diseases;**
- **cause short-circuits of the nervous system;**
- **can have a negative effect on the capacity of the nervous system to provide energy to tissues and organs.**

These three statements are excessively simplistic and lack a contemporary scientific rationale. The first one is in accordance with the mono-causal model, which is not acceptable in modern health science. The second implies that a subluxation can put a stop to the activities of the nervous system, like some sort of a switch. As the nervous system is not an electric system, such parallels between the complexity of the nervous system and electro mechanics cannot be done. The third statement lacks a factual background as the nervous system does not provide energy to tissues and organs.

1. It is possible to detect subluxations before symptoms appear

Today, no evidence supports the theory of ‘subluxation’. There is no objective method to detect ‘subluxation’, hence it would be necessary to show in properly conducted studies that it is possible to detect ‘subluxations’ in asymptomatic persons and, further, to survey these people over time to establish whether asymptomatic people with versus those without subluxations are more likely to develop symptoms. Even if the ‘subluxation’ appears not to be an objective finding, this does not mean that spinal treatment cannot have an effect. The two may not be connected.

**REFERENCES**

1. Bronfort G HM, Evans R, Leininger B, Triano J: **Effectiveness of manual therapies: the UK evidence report**. *Chiropr Osteopat* 2010, **18**:3.

2. Clar C TA, Court R, Lewando Hundt G, Clarke A, Sutcliffe P: **Clinical effectiveness of manual therapy for the management of musculoskeletal and non- musculoskeletal conditions: systematic review and update of UK evidence report**. *Chiropractic & Manual Therapies* 2014, **22**(12).

3. Goncalves G LSC, Leboeuf-Yde C: **Effect of chiropractic treatment on primary or early secondary prevention: a systematic review with a pedagogic approach**. *Chiropr Man Therap* 2018, **26**:10.

4. Battie MC, Videman T, Kaprio J, Gibbons LE, Gill K, Manninen H, Saarela J, Peltonen L: **The Twin Spine Study: contributions to a changing view of disc degeneration**. *Spine J* 2009, **9**(1):47-59.
